# Genomic imprinted genes in reciprocal hybrid endosperm of *Brassica napus*

**DOI:** 10.1186/s12870-021-02908-8

**Published:** 2021-03-16

**Authors:** Hao Rong, Wenjing Yang, Haotian Zhu, Bo Jiang, Jinjin Jiang, Youping Wang

**Affiliations:** 1 Jiangsu Provincial Key Laboratory of Crop Genetics and Physiology, Yangzhou University, Yangzhou, 225009 China; 2 Joint International Research Laboratory of Agriculture and Agri-Product Safety, the Ministry of Education of China, Yangzhou, 225009 China

## Abstract

**Background:**

Genomic imprinting results in the expression of parent-of-origin-specific alleles in the offspring. *Brassica napus* is an oil crop with research values in polyploidization. Identification of imprinted genes in *B. napus* will enrich the knowledge of genomic imprinting in dicotyledon plants.

**Results:**

In this study, we performed reciprocal crosses between *B. napus* L. cultivars Yangyou 6 (Y6) and Zhongshuang 11 (ZS11) to collect endosperm at 20 and 25 days after pollination (DAP) for RNA-seq. In total, we identified 297 imprinted genes, including 283 maternal expressed genes (MEGs) and 14 paternal expressed genes (PEGs) according to the SNPs between Y6 and ZS11. Only 36 genes (35 MEGs and 1 PEG) were continuously imprinted in 20 and 25 DAP endosperm. We found 15, 2, 5, 3, 10, and 25 imprinted genes in this study were also imprinted in *Arabidopsis*, rice, castor bean, maize, *B. rapa*, and other *B. napus* lines, respectively. Only 26 imprinted genes were specifically expressed in endosperm, while other genes were also expressed in root, stem, leaf and flower bud of *B. napus*. A total of 109 imprinted genes were clustered on rapeseed chromosomes. We found the LTR/Copia transposable elements (TEs) were most enriched in both upstream and downstream of the imprinted genes, and the TEs enriched around imprinted genes were more than non-imprinted genes. Moreover, the expression of 5 *AGLs* and 6 pectin-related genes in hybrid endosperm were significantly changed comparing with that in parent endosperm.

**Conclusion:**

This research provided a comprehensive identification of imprinted genes in *B. napus*, and enriched the gene imprinting in dicotyledon plants, which would be useful in further researches on how gene imprinting regulates seed development.

**Supplementary Information:**

The online version contains supplementary material available at 10.1186/s12870-021-02908-8.

## Background

Genomic imprinting is an epigenetic regulation contrary to the classical Mendel’s genetic law, which is reported mainly in plant endosperm and different tissues/organs in mammals [1–3]. Genes with parent-of-origin-specific expression or parentally biased expression are defined as imprinted genes [4, 5]. Thus, the imprinted genes are classified into maternal expressed genes (MEGs) and paternal expressed genes (PEGs). Double fertilization is a specific phenomenon, involves two sperm cells from male gametophyte which were fused with an egg cell and a homodiploid central cell to form a diploid zygote and triploid endosperm, respectively [6]. The triploid endosperm, a seed tissue that does not contribute genetic material to the offspring, has similar function as mammal placenta in providing nutrients for embryo development and seed formation. And it makes the parent-of-origin-specific effects more complicated because of the unbalanced contribution from maternal and paternal genomes [7–9]. Hitherto, the parent conflict theory is the primary explanation for the parent-of-origin-specific effects of imprinted genes on embryo development in plants, but gene imprinting is a complicated phenomenon that some imprinted loci need non-conflict theories [10, 11].

The first imprinted gene *R1* was discovered in maize through genetic trials [12]. But researches on plant imprinted genes were greatly lagged than that in mammals [6, 12–14]. Until 1999, imprinted genes in plants have been gradually identified by the analysis of transcript expressional level, reporter gene activity, or DNA methylation level on alleles that inherited from one specific parent following reciprocal crosses. These genes include *MEA*, *FIS2*, *MPC*, *HDG3/8/9*, *FH5* in *Arabidopsis*, and *FIE1*, *FIE2*, *PEG1*, *MEG1* in maize [9, 15–21]. The high-throughput sequencing technologies greatly facilitated the expression analysis of parental-derived alleles, thus more and more putative imprinted genes have been identified in plants, including *Arabidopsis*, wheat, rice, sorghum, *Capsella rubella* [22–29]. Although most imprinted genes are associated with seed development, but they are not conserved in different plants. Hitherto, a little percentage of imprinted genes were overlapped among different plant species [27, 29]. In addition, it has been proved that imprinted genes primarily influence the nutrient requirements and distribution during endosperm development through dosage effects, which are critical for seed development and vigor [30, 31].

In mammals, approximately 80% of the imprinted genes are clustered on chromosomes, which can usually span millions of bases or more, and the expression of genes nearby can be regulated by these imprinting regulatory regions [32, 33]. The clustered imprinted genes on chromosomes are often regulated by imprinting centers [4]. In plants, most of the imprinted genes are scattered on chromosomes, and only a small number of imprinted genes are clustered on chromosomes [24, 29, 34]. The number of clustered imprinted genes in plants was much less than that in mammals. As reported, the expression of imprinted genes is mainly regulated by DNA methylation and chromatin modification in both plants and mammals, and then the imprinted genes could regulate other gene expression by *cis*- or *trans*-regulation [7, 35]. However, it has also been reported that genomic DNA methylation was not associated with the expression of imprinted genes [29, 36]. Besides, transposable elements (TEs) have been taken as a primary driving force for genomic imprinting, and TEs are extensively identified with hypomethylation on the genome of endosperm, which is important to the seed development [27, 36, 37]. TEs are a large number of repetitive elements on the genome of eukaryotes, which play an important role in plant genome evolution and structural changes [38]. TEs could be activated during hybridization between species and introgression of foreign fragments, and then regulate the downstream gene expression [39]. In castor bean, two types of TEs (LTR/Gypsy and LTR/Copia) were significantly enriched in the vicinity of imprinted genes, and these TEs were extensively demethylated during seed development [27]. Thus, it is speculated that the methylation status of LTR/Gypsy and LTR/Copia TEs might be the main cause of gene imprinting. But this phenomenon was not observed in *Arabidopsis* and maize, which might be due to the specific TE distribution in different plant species [26, 34]. In *A. thaliana*, repression of *AGAMOUS-LIKE* MADS-box genes (*AGLs*) in mutants of imprinted genes were related to the abnormal endosperm cellularization. Down-regulation of genes involved in carbohydrate metabolism (especially genes encode polygalacturonases) would affect pectin hydrolysis in triploid mutants of *A. thaliana* imprinted genes, and finally influence the endosperm cellularization and seed viability [40].

The third largest oil crop in the world, *Brassica napus* L. (AACC, 2n = 38), is an allopolyploid derived from natural hybridization between two diploids *B. rapa* (AA, 2n = 20) and *B. oleracea* (CC, 2n = 18) [41]. Since it is a major resource of edible oil, biofuel, and animal fodder, genetic and epigenetic researches on *B. napus* are important to its breeding course [42]. Comprehensive identification of imprinted gene in *B. napus* will be helpful to elucidate the genetic regulation of seed development. Since the cellularized endosperm in *B. napus* does not proliferate as in monocotyledon crops (e.g. maize, wheat, rice) and castor bean, but gradually disappeared with seed development. It is challenging to collect rapeseed endosperm for genome imprinting analysis [43, 44]. Hitherto, genome imprinting was barely reported in *Brassica*, except for two studies on *B. rapa* and *B. napus*, which identified the putative imprinted genes at one developmental stage of endosperm [45, 46]. Thus, identification of imprinted genes from the reciprocal endosperm of *B. napus*, including different developmental stages, will be of great benefit to the genetic mechanism of genomic imprinting in dicotyledon plants.

In the present study, we performed high-throughput RNA-seq on the endosperm of reciprocal crosses between two *B. napus* cultivars, Zhongshuang 11 (ZS11) and Yangyou 6 (Y6). The maternal and paternal specific single nucleotide polymorphisms (SNPs) were identified for genome-wide screening of imprinted genes. Based on the parent-specific expression, we identified 297 imprinted genes, including 283 MEGs and 14 PEGs. Interestingly, 36 of 297 imprinted genes were continuously imprinted during endosperm development. Only 26 imprinted genes were specifically expressed in endosperm, while other genes were also expressed in other tissues of *B. napus*, rather than endosperm-specific. A total of 109 imprinted genes were clustered on rapeseed chromosomes, and we found the LTR/Copia TEs were most enriched in both upstream and downstream of the imprinted genes. This research provided a more comprehensive identification of imprinted genes in *B. napus*, and enriched the gene imprinting in dicotyledon plants, which would be useful in further researches on how gene imprinting regulates seed development.

## Results

### Transcriptome sequencing and parental specific SNP calling

To distinguish the parental-derived allelic expression in hybrids, we performed the deep high-throughput RNA sequencing on the 20 days after pollination (DAP) and 25 DAP endosperm of ZS11 and Y6 to discover the SNPs between two parents. In total, we obtained 399.78 million paired-end reads, with an average of 35.33, 30.2, 35.46 and 31.97 million reads for 20 DAP endosperm of ZS11 (ZS11_20 DAP), 25 DAP endosperm of ZS11 (ZS11_25 DAP), 20 DAP endosperm of Y6 (Y6_20 DAP), and 25 DAP endosperm of Y6 (Y6_25 DAP), respectively. These reads were mapped to 61,278, 60,174, 60,604 and 59,801 genes of *B. napus* with fragments per kilobase per million (FPKM) > 1 in the endosperm of ZS11_20 DAP, ZS11_25 DAP, Y6_20 DAP, Y6_25 DAP, respectively. The three biological replicates of RNA-seq data were confirmed with personal correlation coefficient values of *R* = 0.96 ~ 0.99 (Additional file 1: Fig. S1). The SNPs of ZS11 and Y6 were identified in comparison to the *B. napus* (European winter oilseed cultivar ‘Darmor-*bzh*’) reference genome using Hisat2 and Samtools. Based on the 91.22, 93.03, 92.95 and 92.96% reads in ZS11_20 DAP, ZS11_25 DAP, Y6_20 DAP, and Y6_25 DAP mapped to the reference genome, the uniquely mapped reads were retained for stringent SNP screening, only the homozygous SNPs identified in at least two biological replicates and covered by ≥10 reads in each sequencing library were kept for further analysis. A total of 35,928 and 28,775 SNPs between ZS11 and Y6 were identified in 20 DAP and 25 DAP endosperms, respectively. These SNPs covered 15,738 and 13,721 genes, of which, 10,672 genes were overlapped in the 20 DAP and 25 DAP endosperm (Additional file 2: Table S1; Additional file 3: Table S2). Similarly, the uniquely mapped reads in the endosperm of reciprocal hybrids (7.55 and 5.57 million reads of 20 and 25 DAP hybrid endosperm) were used for SNP identification, and the reads with paternal or maternal specific SNPs were extracted for allelic expression analysis (Additional file 4: Table S3; Additional file 5: Table S4).

### Genome-wide identification of imprinted genes in hybrid endosperm

**Fig. 1:**
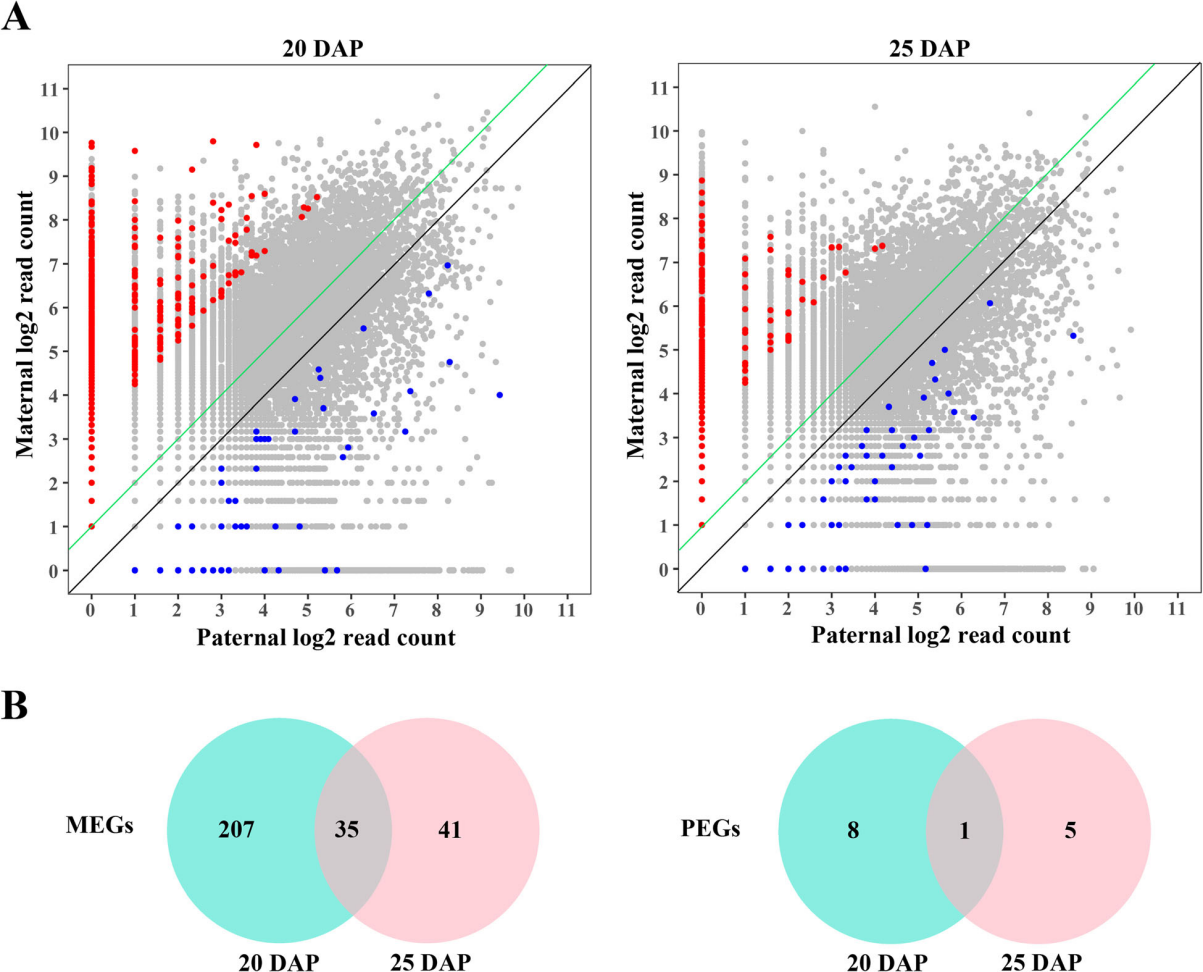
Allele-specific expression analysis of genes in reciprocal endosperm. **a**. Log_2_ normalized read counts for all SNP loci for the endosperms. The red dots represent the MEG loci sorted out with > 90% read percentage bias and the blue dots represent the PEG loci sorted out with > 60% read percentage bias in both reciprocal cross endosperms (*q* < 0.05). The black line and green line denoted the 1:1 and 2:1 ratios (maternal: paternal), respectively. **b**. Venn diagram of MEGs and PEGs between 20 and 25 DAP endosperm. MEG, maternal expressed gene. PEG, paternal expressed gene. DAP, day after pollination

Based on the ratio of maternal-derived and paternal-derived reads at each SNP loci, we used stringent criteria to screen the imprinted genes that MEGs should obey to allelic expression ratio of maternal: paternal ≥10: 1 (5 times of maternal: paternal = 2: 1, ≥ 90% maternally biased expression), and PEGs were screened with a ratio of paternal: maternal ≥3: 2 (3 times of paternal: maternal = 1: 2, ≥ 60% paternally biased expression) in both reciprocal hybrid endosperm (Additional file 4: Table S3; Additional file 5: Table S4). The genes with parental biased expression in three biological replicates of reciprocal hybrid endosperm were identified as imprinted genes (*q* < 0.05). We found that the majority of these genes obeyed the ratio of maternal: paternal = 2: 1 in both 20 DAP and 25 DAP endosperm, only 1.132% genes exhibited parent-of-origin differences in allelic expression (χ^2^ test, *q* < 0.05) (Fig. 1a). In total, we identified 251 imprinted genes (242 MEGs and 9 PEGs) in 20 DAP endosperm and 82 imprinted genes (76 MEGs and 6 PEGs) in 25 DAP endosperm (Additional file 6: Table S5; Additional file 7: Table S6). Only 36 genes (35 MEGs and 1 PEG) were continuously imprinted in 20 and 25 DAP endosperm. The remaining 261 genes were imprinted in different stages of endosperms, including 215 genes imprinted in 20 DAP endosperm (207 MEGs and 8 PEGs) and 46 genes imprinted in 25 DAP endosperm (41 MEGs and 5 PEGs) (Fig. 1b; Additional file 8: Table S7). This indicated that most imprinted genes exhibited inconsistent expression pattern at different developmental stages of endosperm in *B. napus*. Among the stage specific imprinted genes, 73 genes were imprinted in one stage but exhibited bi-allelic expression pattern in another developmental stage of hybrid endosperm. The rest 118 genes were imprinted in only one stage, or imprinted in two developmental stages but supported by less than 10 reads in one stage. Moreover, we found 8 pairs of homologous genes imprinted in A and C subgenome (*BnaA05g00640D* and *BnaC04g51420D*, *BnaA06g07630D* and *BnaC05g09100D*, *BnaA10g22530D* and *BnaC03g49920D*, *BnaA08g18690D* and *BnaC03g58190D*, *BnaA03g21790D* and *BnaC03g26060D*, *BnaA10g19500D* and *BnaC09g54550D*, *BnaA09g42740D* and *BnaC08g35220D*, *BnaA01g23480D* and *BnaCnng44170D*). As to the remaining 281 unpaired genes, 200 genes (71%) were imprinted on the A subgenome and 81 genes (29%) were imprinted on the C subgenome. This indicated that most of the imprinted genes were biased to A subgenome.

### Experimental validation of candidate imprinted genes in *B. napus*

**Fig. 2:**
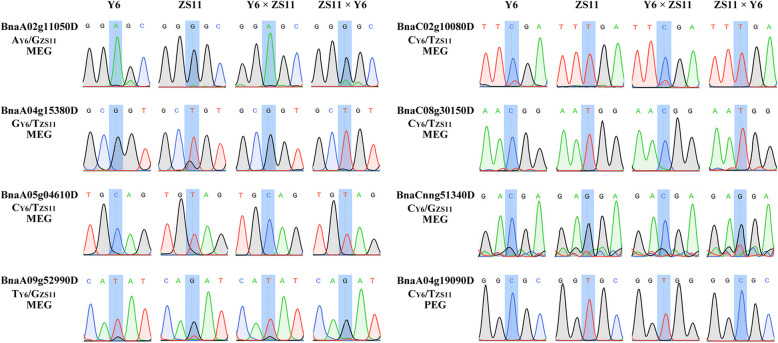
Validation of imprinted genes by RT-PCR sequencing

In order to confirm the imprinted genes in *B. napus*, the allele-specific expression analysis was carried out on randomly selected genes (7 MEGs and 1 PEG). The amplified RT-PCR fragments of endosperms from reciprocal crosses and self-pollinated parents were sequenced to identify the parental SNPs. Consistent with the SNPs identified by RNA-seq data, all the 8 genes were confirmed with ZS11 or Y6 specific SNPs in the hybrid endosperm, indicating these genes with parent-of-origin expression patterns (Fig. 2). For example, the MEGs (*BnaA02g11050D*, *BnaA04g15380D*, *BnaA05g04610D*, *BnaA09g52990D*, *BnaC02g10080D*, *BnaC08g30150D*, *BnaCnng51340D*) were identified with maternal specific SNPs in both reciprocal crosses. While a PEG (*BnaA04g19090D*) was confirmed with paternal specific SNP in both reciprocal crosses.

To know whether these imprinted genes identified in *B. napus* were conserved in other plant species, we blasted their homologous gene pairs in other species, and found 15, 2, 5, 3, 10, and 25 imprinted genes in this study were also imprinted in *Arabidopsis* [22, 23], rice [24], castor bean [27], maize [25], *B. rapa* [45], and other *B. napus* lines [46], respectively (Table 1). Interestingly, most of the overlapped genes were MEGs (except for three PEGs overlapped with *B. rapa*), and the imprinted genes in *B. napus* were more conserved with other dicots than monocots. We found the homologs of *BnaA06g38220D* were also imprinted in rice and maize. This agreed to the previous report that gene imprinting is poorly conserved among different species [27].

**Table 1:** Overlaps between the rapeseed imprinted genes and those of *Arabidopsis*, rice, castor bean, maize, and other *Brassicas*

	Query	Subject	Identity	Mapping length	Mismatch	Gap	E value
*Arabidopsis*	*BnaA01g15540D*	*AT4G26420*	76.47	652	90	6	0
	*BnaA03g27380D*	*AT3G01640*	90.29	264	95	3	1.00E-110
	*BnaA03g42520D*	*AT4G16380*	75.67	248	20	1	6.00E-148
	*BnaA04g19750D*	*AT2G33770*	81.26	122	59	1	2.00E-36
	*BnaA05g08350D*	*AT2G35670*	46.67	744	264	9	0
	*BnaA05g27940D*	*AT3G11000*	56	547	42	3	0
	*BnaA06g05440D*	*AT1G09540*	65.96	290	23	1	0
	*BnaA07g33830D*	*AT1G77960*	38.12	669	308	10	7.00E-178
	*BnaA08g26320D*	*AT1G09540*	83.15	629	47	3	0
	*BnaA09g01980D*	*AT3G27300*	92.25	580	49	2	0
	*BnaA10g22530D*	*AT5G64260*	78.43	306	30	1	5.00E-162
	*BnaC05g48510D*	*AT3G02110*	90.7	473	32	2	0
	*BnaC07g22140D*	*AT2G03210*	86	313	33	0	0
	*BnaC09g20470D*	*AT2G01300*	82.05	422	30	1	0
	*BnaCnng40210D*	*AT3G16720*	68.52	578	191	9	0
*B. rapa*	*BnaA01g08850D*	*Bra013274*	85.08	496	46	2	0
	*BnaA04g19090D*	*Bra005543*	57.7	461	84	2	2E-123
	*BnaA05g08350D*	*Bra005316*	50.26	191	14	4	4E-47
	*BnaA05g08770D*	*Bra005362*	59.74	688	201	1	0
	*BnaA06g05440D*	*Bra020016*	84.77	243	14	0	2E-108
	*BnaA06g37970D*	*Bra019964*	87.5	392	22	1	0
	*BnaA08g26320D*	*Bra020016*	82.34	368	24	3	2E-151
	*BnaA09g13800D*	*Bra027091*	65.84	363	17	5	3E-109
	*BnaAnng03730D*	*Bra036061*	82.27	141	8	1	7E-62
	*BnaC02g44940D*	*Bra007859*	78.81	387	32	3	0
Rice	*BnaA06g38220D*	*LOC_Os03g01320.1*	76.19	84	20	0	7E-39
	*BnaA09g13800D*	*LOC_Os07g37620.1*	49.03	155	63	5	7E-33
Castor bean	*BnaA06g34330D*	*29,780.m001362*	68.1	232	45	3	5E-109
	*BnaC05g09100D*	*29,792.m000624*	50.21	482	234	5	2E-174
	*BnaA08g21920D*	*30,170.m014165*	46.04	556	210	17	3E-108
	*BnaC04g34200D*	*29,905.m000439*	39.93	278	149	7	3E-50
	*BnaC07g36310D*	*27,837.m000165*	66.43	143	48	0	1E-42
Maize	*BnaA06g38220D*	*GRMZM2G379898*	74.12	85	22	0	4E-38
	*BnaA04g15380D*	*GRMZM2G445602*	70.26	548	142	5	0
	*BnaA08g16960D*	*GRMZM2G108032*	56.93	476	199	3	0
*B. napus(Rapeseed lines YN171 and 93275)*	*BnaA02g15580D*	*BnA02g0069250*	99.02	509	5	0	0
	*BnaA02g34950D*	*BnA02g0050760*	99.42	344	2	0	0
	*BnaA03g24210D*	*BnA09g0353510*	93.07	231	15	1	1E-156
	*BnaA03g31730D*	*BnC03g0579390*	85.71	385	53	1	0
	*BnaA03g48600D*	*BnA03g0151360*	100	173	0	0	4E-125
	*BnaA04g24790D*	*BnA04g0183470*	99.03	516	5	0	0
	*BnaA06g07630D*	*BnA06g0231270*	98.8	502	6	0	0
	*BnaA06g26050D*	*BnA06g0253460*	100	405	0	0	0
	*BnaA07g18120D*	*BnA03g0127550*	99.61	507	2	0	0
	*BnaA07g33830D*	*BnA07g0301490*	100	204	0	0	2E-149
	*BnaA08g06260D*	*BnA08g0311780*	99.68	317	1	0	0
	*BnaA08g25430D*	*BnA08g0332430*	97.99	398	8	0	0
	*BnaA08g26320D*	*BnA08g0333300*	97.4	346	8	1	0
	*BnaA08g29710D*	*BnA08g0311890*	98.42	506	8	0	0
	*BnaA09g31650D*	*BnA09g0368910*	99.7	337	1	0	0
	*BnaA10g23330D*	*BnA10g0420100*	98.83	511	2	1	0
	*BnaA10g23660D*	*BnA10g0419770*	99.67	307	1	0	0
	*BnaAnng03730D*	*BnA09g0368240*	98.77	162	2	0	3E-113
	*BnaAnng03940D*	*BnA09g0368020*	98.41	504	7	1	0
	*BnaC03g33640D*	*BnC05g0703810*	100	141	0	0	1E-97
	*BnaC04g00750D*	*BnC04g0620020*	100	254	0	0	0
	*BnaC08g34600D*	*BnC08g0874770*	100	242	0	0	5E-156
	*BnaC08g35220D*	*BnC08g0875510*	98.91	275	3	0	0
	*BnaC09g20470D*	*BnUnng1014900*	93.46	153	7	1	2E-99
	*BnaC09g54550D*	*BnC09g0927560*	100	322	0	0	0

### Functional characterization of imprinted genes in *B. napus*

**Fig. 3:**
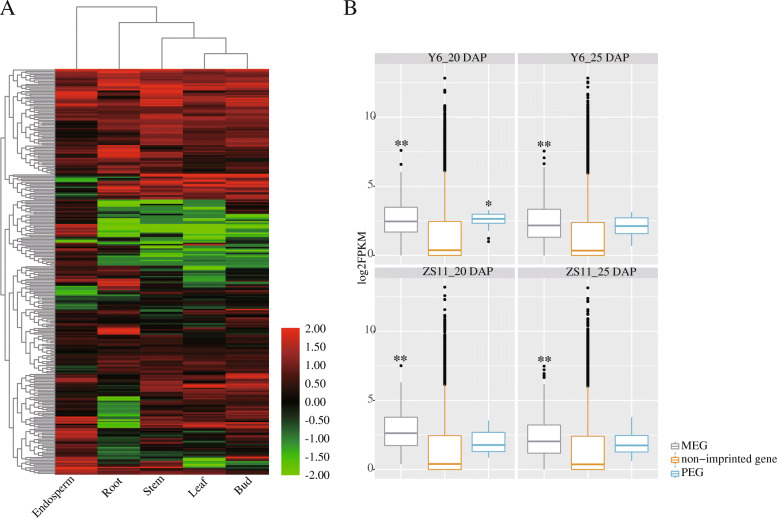
Expression pattern of imprinted genes. **a**. Heat map of imprinted genes in endosperm, root, stem, leaf and bud (log_10_FPKM). **b**. Box-plot of expression of imprinted genes and non-imprinted genes in 20 DAP and 25 DAP endosperm of parents (log_2_FPKM). Student’s *t-test* was used for statistical analysis between imprinted genes and non-imprinted genes (*, *p* ≤ 0.05; **, *p* ≤ 0.01)

As reported in other plants, imprinted genes were mostly identified in endosperm, and with endosperm-specific or endosperm-preferred expression patterns [22, 24, 47]. Here, we analyzed the expression pattern of rapeseed imprinted genes in different tissues of *B. napus* cv. ZS11, including root, stem, leaf, bud and endosperm (Fig. 3a; Additional file 9: Table S8). Inconsistent with previous reports, we only found 26 rapeseed imprinted genes were specifically expressed in endosperm. While other imprinted genes were also expressed in root, stem, leaf and flower bud of *B. napus*, rather than endosperm-specific. Thus, we may suspect that most of the imprinted genes have functions in the development of rapeseed, not only with specific effects on the endosperm development. In addition, we also compared the expression of imprinted genes and non-imprinted genes in the parent endosperm. The results showed that the imprinted genes were highly expressed than non-imprinted genes in both 20 DAP and 25 DAP endosperm. Besides, the expression of MEGs in parent endosperm was higher than that of PEGs (Fig. 3b).

**Fig. 4:**
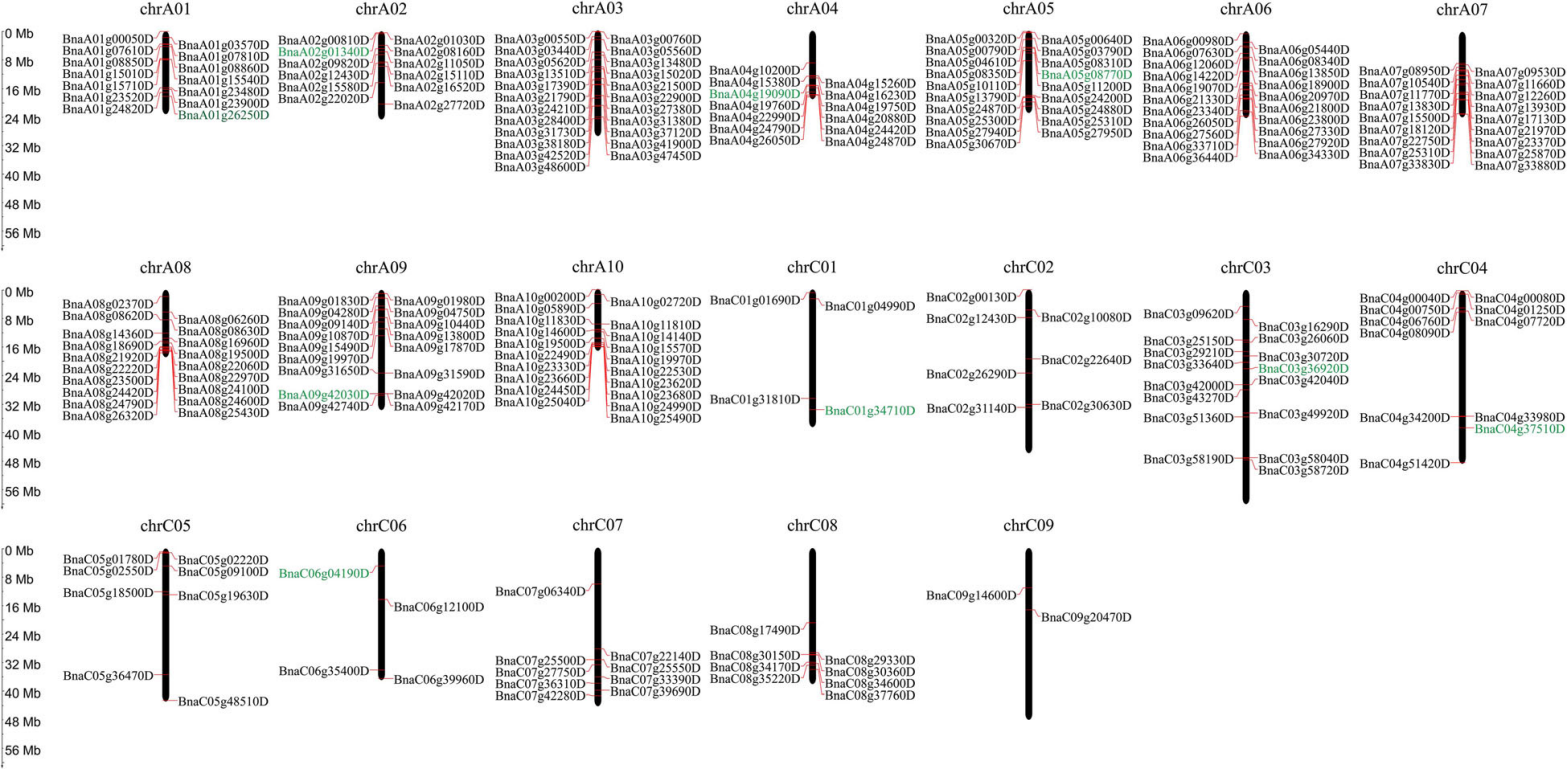
The distribution of imprinted genes on the *B. napus* chromosomes. MEGs in black font and PEGs in green font

**Fig. 5:**
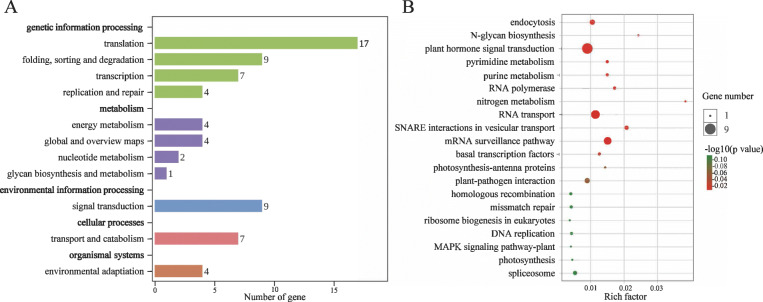
KEGG enrichment of imprinted clusters. **a**. KEGG pathway analysis of genes within the cluster 24/25/28/29. **b**. Top 20 pathways enriched with genes in the cluster 24/25/28/29. Rich factor means the ratio of imprinted gene number to transcript number in each KEGG pathway

**Fig. 6:**
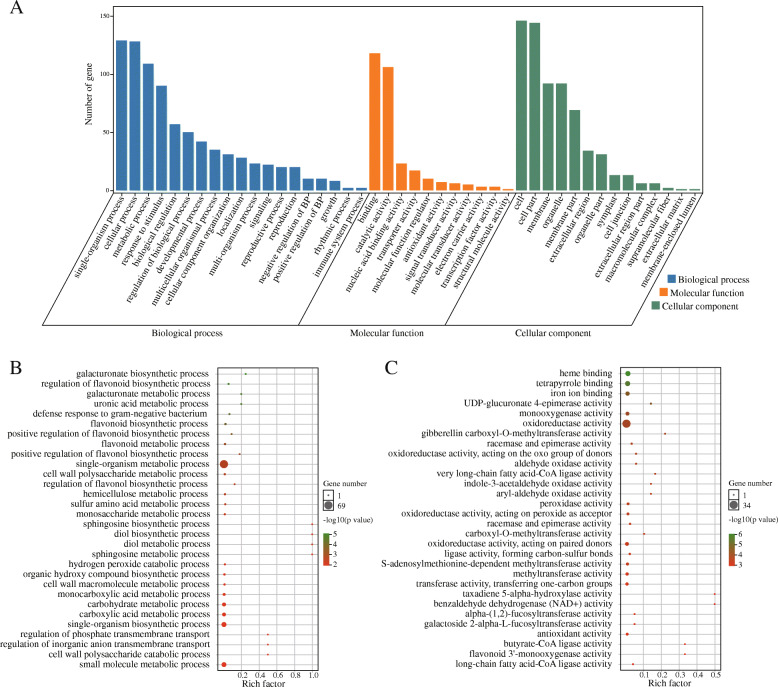
GO enrichment analysis. **a**. GO enrichment analysis of imprinted genes. **b**. Top 30 GO terms enriched in biological process. **c**. Top 30 GO terms enriched in molecular function. Rich factor means the ratio of imprinted gene number to transcript number in each GO term

In mammalian, most of the imprinted genes are clustered on the chromosomes, and their expression is regulated by imprinted regulatory regions [48]. But in plants (e.g. *Arabidopsis*, rice, sorghum, and castor bean), only a few imprinted genes are clustered on the chromosome. Here, we mapped 245 imprinted genes (except for 47 MEGs and 5 PEGs scattered to Ann_random or Cnn_random chromosomes) to the 19 chromosomes of *B. napus*. Altogether, we identified 35 clusters that unevenly distributed on the A and C subgenome (Fig. 4; Additional file 10: Table S9), including 29 clusters (92 imprinted genes) on A genome and 6 clusters (17 imprinted genes) on C genome. These clustered genes might be controlled by regional regulations. Besides, we found five largest clusters, including cluster 16 (5 imprinted genes) on the end of A05, cluster 24 (5 genes) and cluster 25 (6 genes) on A08, cluster 28 (5 genes) and cluster 29 (5 genes) on A10. Kyoto Encyclopedia of Genes and Genomes (KEGG) analysis on genes nearby the cluster 24/25/28/29 revealed that most genes were involved in transcription, translation, energy metabolism, glycan biosynthesis and metabolism, transport and catabolism, plant hormone signal transduction, and environmental adaptation (Fig. 5). Gene ontology (GO) enrichment analysis revealed that most imprinted genes were assigned to biological functions (cellular process, single-organism process, metabolic process, response to stimulus and biological regulation), molecular functions (binding and catalytic activity), cellular components (cell and cell part) (Fig. 6a; Additional file 11: Table S10). In triploid *adm* and *suvh7* mutants of *A. thaliana*, the seed rescue was strongly correlated with decreased expression of *AGLs*, which may further affect endosperm cellularization and cause embryo arrest. Suppression of genes related to pectin hydrolysis in these mutants were also related to the abnormal endosperm cellularization and seed viability [40]. Among the top enriched GO terms, we found galacturonate biosynthetic and metabolic process, cell wall polysaccharide and macromolecule metabolic process, UDP-glucuronate 4-epimerase activity, and alpha-(1,2)-fucosyltransferase activity were enriched (Fig. 6b, c). This indicated that many imprinted genes were involved in the cell wall biosynthesis and related to endosperm cellularization. In rapeseed, endosperm accumulated and cellularized until 18 to 32 DAP, which was then disappeared after transferring nutrients to embryos [43]. Thus, imprinting of these genes related to cell wall biosynthesis would affect pectin hydrolysis, endosperm cellularization and seed viability. Further molecular functional studies on these imprinted genes would enrich our knowledge in seed development of *B. napus*.

### Transposable element enrichment around imprinted genes

**Fig. 7:**
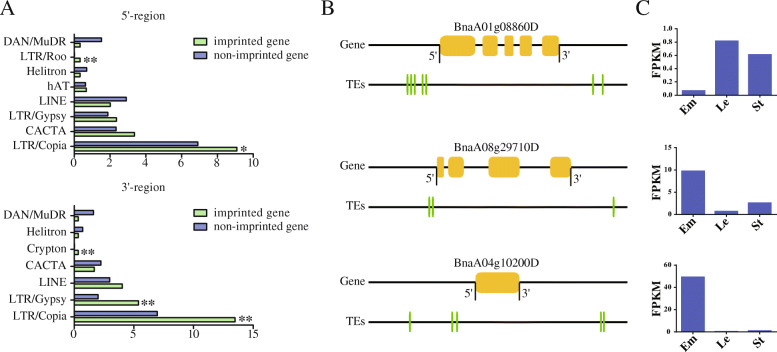
Type of TEs in vicinity of imprinted genes. **a**. Type of TEs in 5′- and 3′-region of imprinted genes and non-imprinted genes. X-axis is the number of transposons in 5′- and 3′-region per 100 genes. **b**. TE position in 5′- and 3′-region of imprinted genes. The yellow boxes represent gene body regions, and the green boxes represent TEs. **c**. Expression level of imprinted genes in endosperm (Em), leaf (Le) and stem (St). χ^2^ test was performed for statistical analysis between imprinted genes and non-imprinted genes (*, *p* ≤ 0.05; **, *p* ≤ 0.01)

In *Arabidopsis* endosperm, the extensively demethylated TEs have been taken as a main driving force of gene imprinting [36, 49]. Therefore, we investigated the number and type of TEs within 5 kb upstream and downstream of the imprinted and non-imprinted genes in *B. napus*. We found the number of TEs enriched around imprinted genes was significantly higher than that around non-imprinted genes. And the TE types enriched in 5′-region were more than that in 3′-region of the imprinted gene (Fig. 7a). Interestingly, we found the LTR/Copia TEs were enriched in both upstream and downstream of the imprinted genes. This was similar to the TE enrichment around imprinted genes in castor bean, which were enriched with LTR/Copia and LTR/Gypsy type TEs. But DNA/MuDR type TEs were enriched around imprinted genes in *Arabidopsis* and *C. rubella*, while CACTA type TEs were enriched around imprinted genes in maize [26–28, 34]. In addition, we analyzed the imprinted genes with TEs enriched in both 5′- and 3′-regions. We found 5′- and 3′- region of *BnaA01g08860D* was enriched with 5 and 2 TEs, respectively. The expression level of *BnaA01g08860D* in endosperm was much lower than that in other tissues. The 5′-region of *BnaA08g29710D* and *BnaA04g10200D* contain 2 and 3 TEs, and the 3′-region contain 1 and 2 TEs, respectively. *BnaA08g29710D* and *BnaA04g10200D* were highly expressed in endosperm than in other tissues (Fig. 7b, c). We suspected that the TEs enriched around these imprinted genes might affect their expression pattern.

### Expression analysis of *AGLs* and genes related to pectin degradation

**Fig. 8:**
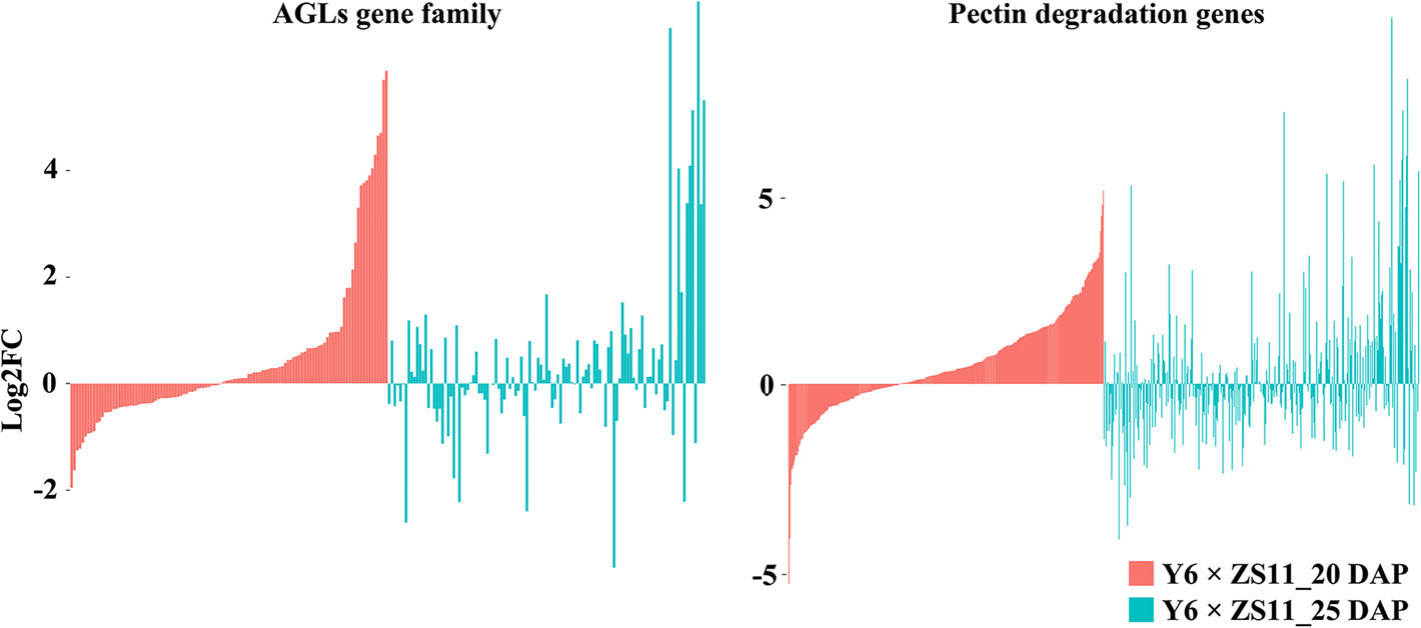
Expression analysis of *AGLs* and pectin degradation pathway genes in reciprocal endosperm

As reported in *A. thaliana*, the expression level of *AGLs* and genes involved in carbohydrate metabolism (e.g. genes encode polygalacturonases) affected endosperm cellularization and pectin hydrolysis in triploid mutants of *A. thaliana* imprinted genes [40, 50]. Since some imprinted genes in rapeseed were also enriched with GO terms related to cell wall synthesis, here we analyzed the expression of 113 *AGLs* and the 189 genes involved in pectin degradation pathway in the reciprocal hybrid endosperm (Fig. 8). Comparing with two parents, we found the expression of 5 *AGLs* (*BnaC02g01970D*, *BnaA03g29530D*, *BnaA06g12900D*, *BnaC02g40410D* and *BnaC01g28010D*) were changed with |log_2_fold change| > 5 in hybrid endosperm, and the expression of most *AGLs* were not significantly changed. As to the genes involved in pectin degradation pathway, we found the expression of *BnaC08g30060D*, *BnaA09g16050D*, *BnaC04g24110D*, *BnaA01g04630D*, *BnaC01g06140D* and *BnaA06g16310D* were significantly changed in hybrid endosperm compared with that in parent endosperm.

## Discussion

Genomic imprinting is an epigenetic phenomenon against to the classical Mendel’s law. Hitherto, imprinted genes have been identified in several plants, including *Arabidopsis* (341 genes), rice (262 genes), maize (356 genes), castor bean (209 genes), and wheat (372 genes) [23–25, 27, 47]. Functional annotation of these imprinted genes revealed that they are mainly enriched in the regulation of pigmentation, protein storage, transcriptional regulation, catalysis and chromatin modification [26]. However, the functional studies on imprinted genes are barely reported, which should be crucial for genetic regulation on plant development, especially seed development [15, 51–53]. *B. napus* is the third largest oil crop with great economic values, but only one report on genome imprinting of 30 DAP endosperm is available now [46]. Research on gene imprinting of different *B. napus* cultivars will broaden our understanding of seed development, and lay a basis for genetic breeding of rapeseed with high-yield and high-quality.

### Genomic imprinting pattern in endosperm of *B. napus*

In this study, we generated transcriptome sequencing data of developing endosperm from two *B. napus* cultivars (Y6 and ZS11) and their reciprocal hybrids. Based on a stringent criteria for screening of genes with paternal or maternal-specific SNPs, we identified 297 imprinted genes in hybrid endosperm, including 283 MEGs and 14 PEGs. The number of PEGs in *B. napus* was much less than MEGs, which is consistent with reports in *A. thaliana*, maize, sorghum and *A. lyrata* [29, 34, 54, 55]. The unbalanced MEGs and PEGs in plants agreed with the maternal-offspring co-adaptation theory, indicating the maternal genes were more favored during natural selection [56]. The different number of imprinted genes in 20 and 25 DAP rapeseed endosperm agreed with the previous report that imprinted genes varies with plant development. Xin et al. found the number of MEGs maximized in 10 DAP endosperm of maize, while the number of PEGs peaked in 7 DAP endosperm [57]. In wheat, 47.3% genes exhibited consistent imprinted expression pattern in 15, 20, and 25 DAP endosperm [47]. Besides, 40% genes were consistently imprinted in 10 and 12 DAP endosperm of maize [26, 54]. The percentage of consistently imprinted genes in this manuscript was much lower than that in wheat and maize. Since the imprinted genes in *Brassica* has not been well studied, identification of genome imprinting in more developmental stages of endosperm from different cultivars would be helpful to elaborate the imprinting characteristics in *Brassica*. Among the genes imprinted in both 20 and 25 DAP endosperm, we found *BnaA03g05560D* is a homolog of *OsCWA1/BC1* that encodes a COBRA protein. OsCWA1/BC1 is important in secondary cell wall biosynthesis [58]. *BnaA05g04610D* is a homolog of *AtPME17*, which can change pectin physicochemical properties, induce the reduction of galacturonic acid to modify the cell wall architecture [59]. *BnaC05g18500D* is a homolog of *AtPME6*, which encodes a pectin methyl esterase that involved in pectin metabolism of cell wall, and embryo cell expansion and development [60, 61].

### Bias of genome imprinting in A and C subgenome of *B. napus*

*B. napus*, as an allotetraploid could provide a model to determine whether genome imprinting exhibits expression bias between the homoeologous from A and C subgenome. Among the 297 imprinted genes in *B. napus*, we found 14 pairs of homologs, but only 8 homologous pairs were located on the A and C subgenome, respectively. As to the remaining 281 unpaired genes, 200 genes (71%) were imprinted on the A subgenome and 81 genes (29%) were imprinted on the C subgenome. This indicated that most of the imprinted genes were biased to A subgenome. During the polyploidization of *B. napus*, Li et al. found that the majority of gene pairs (~ 86.7%) in *B. napus* maintained their expression pattern in two diploid progenitors, and approximately 78.1% of the gene pairs showed expression bias with a preference toward the A subgenome [62]. Wu et al. also showed that ~ 36.5% of the expressed gene pairs in resynthesized *B. napus* displayed expression bias with a slight preference toward the A genome [63]. Thus, we may suspect that the bias of imprinted gene number might be related to the expressional bias during rapeseed polyploidization.

### Genome imprinting is not conserved among different species

Studies on *Arabidopsis*, rice, maize and other plants confirmed that imprinted genes are not conserved among plant species, only 21 imprinted genes were conserved between *Arabidopsis* and *C. rubella*, two genes were imprinted in both rice and *Arabidopsis* [28, 29]. In the present study, we found 15, 2, 5, 3, 10, and 25 rapeseed imprinted genes with homologs imprinted in *Arabidopsis*, rice, castor bean, maize, *B. rapa*, and other *B. napus* lines, respectively [22–27]. The little overlap of imprinted genes among species, even between different lines of same species is common in plants, which might be due to the filtering of data analysis. In *Arabidopsis*, only 19 genes were imprinted both in *Ler* and *Col-0* accessions, while large majority of genes (81%) were unique to a single study [64]. In the four available studies of maize, the majority of the imprinted genes (65% MEGs and 41% PEGs) were proposed by a single study, only 14 MEGs (8%) and 23 PEGs (13%) were commonly identified by all four studies [64]. Here, we only found 10 and 25 genes with orthologous imprinted in *B. rapa* and other *B. napus* lines (YN171 and 93275), respectively [45, 46]. Among the conserved imprinted genes among species, *BnaA06g38220D* was found with homologs imprinted in maize and rice, which encodes an extensin-like protein and might be involved in lignin biosynthesis and interspecific reproductive disorder [65, 66]. The homologs of *BnaC07g22140D* in *A. thaliana* (*AT2G03210*) encodes xyloglucan fucosyltransferase 2 (FUT2) that might be participated in cell wall organization [67]. *BnaA05g08350D* is a homolog of *AtFIS2*, which is important in repressing seed development before fertilization, and regulates embryo and endosperm development after the double fertilization [16, 68]. In this study, we found 10 genes with homologs imprinted in *B. rapa,* which is a diploid parent of *B. napus*. These genes were annotated with functions in regulating DNA methylation (*BnaA05g08770D* and *BnaA05g08350D*) and cellulose synthesis (*BnaA0826321D* and *BnaA08g26320D*) (Additional file 6: Table S5; Additional file 7: Table S6). Hitherto, there is only one report of genome imprinting in 30 DAP endosperm of *B. napus* [46], and 25 imprinted genes (8.42%) were overlapped with our study. These genes were annotated with functions in regulating ovule development (*BnaA03g24210D*), cell wall modification (*BnaA04g24790D*), apoptosis (*BnaA10g23660D*), and response to oxidative stress (*BnaC09g54550D* and *BnaA09g31650D*) (Additional file 6: Table S5; Additional file 7: Table S6). *BnaA0826321D* is a homolog of *OsMYB61* and imprinted in *B. rapa* and other *B. napus* lines. In rice, OsNAC29/31 directly activate OsMYB61, which in turn activates the expression of cellulose synthase genes, and finally regulates secondary wall cellulose synthesis [69]. *BnaA05g08770D*, an overlapped imprinted gene with *B. rapa*, is a homolog of *AtSUVH5*. AtSUVH5 interacts with AtHDA19 and negatively regulates seed dormancy [70].

### The expressional pattern and clustering of imprinted genes in *B. napus*

Previous researches reported that 80% of imprinted genes in *Arabidopsis*, 70% in sorghum, and 78% in rice were endosperm-specific or endosperm preferentially expressed genes. However, only 40% of imprinted genes in castor bean were endosperm-specific genes [24, 27, 29, 34]. In the present study, we found 26 imprinted genes were endosperm-specific expressed genes, while other genes were also expressed in different tissues of *B. napus*, indicating they might play functions throughout rapeseed development, not only in seed development.

In mammals, most of the imprinted genes are clustered on the chromosome [32, 33, 48]. In plants, only 28 imprinted genes were assigned to 12 clusters in sorghum, 77 genes in 33 clusters were identified in maize, and 7 imprinted genes in 3 clusters were reported in castor bean [26, 27, 29]. In rapeseed, we found 109 imprinted genes assigned to 35 clusters on chromosomes. The chromosome regions around these clusters might be imprinting control regions and could influence imprinted gene expression. We also found homologous gene pairs in these clusters, suggesting that genome duplication also made gene imprinting more complicate in rapeseed [34].

### Functions of imprinted genes in regulating endosperm development

Imprinted genes have been confirmed with important roles in endosperm development [40, 50]. Here we found the rapeseed imprinted genes were also enriched in process related to cell wall biosynthesis, and speculated that they might be related to the endosperm development. For example, *BnaA03g05560D*, which encodes irregular xylem 6, was enriched in plant-type secondary cell wall biogenesis (GO: 0009834). Its homologous gene in rice is crucial for assembly of secondary cell wall [58]. *BnaA01g03570D* was enriched in cell wall organization (GO: 0071555), and the homolog in *Arabidopsis* was required for branch extension of xylan in cell wall [71]. In addition, most of the imprinted genes were enriched with binding and catalytic function (e.g. hydrolase and transferase activities). We also found imprinted genes enriched in biological processes, such as carbon metabolism, cellular metabolism, and biosynthesis of biological macromolecules. In *Arabidopsis*, triploid seed abortion in mutants of imprinted genes were related to the expression of genes involved in cell wall synthesis and degradation in endosperm [40, 50]. Among the *B. napus* imprinted genes identified in this study, *BnaA05g04610D*, *BnaC05g18500D* and *BnaA04g24790D* were involved in pectin metabolism, which might be involved in endosperm cellularization. Besides, the expression changes of *AGLs* can hinder the formation and degradation of endosperm cell wall, and finally affect endosperm cellularization and lead to seed abortion. Here, we only found the expression of 5 *AGLs* was significantly changed in hybrid endosperm compared with parent endosperm. Of which, *BnaA03g29530D* (a homolog of *AtAGL91*) and *BnaA09g16050D* were significantly changed in 20 and 25 DAP endosperm of reciprocal hybrids. In *Arabidopsis*, the spatial-temporal expression of *AGL91* was regulated by maternal siRNAs, and disruption or overexpression of *AGL91* in the endosperm altered seed size [72]. *BnaA09g16050D* encodes a pectin lyase-like superfamily protein, which might be a pectic substance that occurred as structural polysaccharides in the middle lamella and primary cell walls of higher plants [73]. *BnaC04g24110D* also encodes a pectin lyase-like superfamily protein, and its homologs in *Arabidopsis* (*ADPG1*) regulates lignin content and composition [74]. *BnaA05g04610D* is a homolog of *AtPME17*, which can change pectin physicochemical properties, induce the reduction of galacturonic acid to modify the cell wall architecture [75]. *BnaAnng26700D* is a homolog of *OsFIE1*, which is an essential member of polycomb repressive complex 2 (PRC2) that plays vital roles in early seed development through regulating endosperm cellularization and seed size [76, 77].

### Putative regulation of imprinted genes in *B. napus*

TEs are important in driving plant genome expansion and species evolution, through influencing the genome structure (gene structure, inversion, translocation, and recombination) and gene expression [78–82]. It has been reported that TEs enriched in the vicinity of imprinted genes were extensively demethylated in endosperm, suggesting that TEs might be a driving force of genome imprinting [27]. In the present study, we also found significant enrichment of LTR/Copia and LTR/Gypsy TEs in the upstream and downstream of the imprinted genes. This was similar to castor bean, but the type of TEs enriched around imprinted genes of *Arabidopsis*, maize, and *C. rubella* is different [26–28, 34].

Genomic DNA methylation has also been taken as a driving force of genomic imprinting in plants, and most differentially methylated regions (DMRs) identified were hypomethylated in maternal alleles and hypermethylated in paternal alleles [26, 27]. However, only 11 imprinted genes (0.06%) in *B. napus* were identified with DMRs, including one MEG confirmed with high GC methylation [46]. In the present study, DNA methylation analysis was not performed. But we correlate the imprinted genes with the miRNA regulation of *B. napus* [83], and found 12 imprinted genes might be targeted by miR158, miR171, miR160, miR399, miR394, and other six novel miRNAs. In *Arabidopsis*, functional loss of miR171 caused abnormal embryogenesis, and it was proved that a correct relationship between miR171 and HAM1 is necessary for normal embryogenesis [84]. miR160, miR171, miR394, and miR399 may participate in the early embryonic development and morphogenesis of maize and *Arabidopsis*, through transcriptional regulation of their target genes [85, 86]. In addition, miR160 controls somatic embryogenesis induction by negatively regulating auxin-related genes (*ARF10*, *ARF16*, and *ARF17*) [87].

## Conclusions

In the present study, we identified a total of 297 imprinted genes, including 283 MEGs and 14 PEGs in reciprocal hybrid endosperm, basing on the specific SNPs in two *B. napus* cultivars (Y6 and ZS11). Only 36 genes were continuously imprinted in 20 and 25 DAP endosperm. Besides, many imprinted genes in rapeseed were annotated to GO terms related to cell wall biosynthesis and endosperm cellularization. TEs analysis nearby the imprinted and non-imprinted genes revealed that LTR/Copia TEs were most enriched, indicating they might influence the expression pattern of imprinted genes. Moreover, the expression of 5 *AGLs* and 6 pectin-related genes in hybrid endosperm were significantly changed when comparing with that in parent endosperm, which would be helpful to explain the normal developed reciprocal hybrid seeds. Generally, identification and characterization of imprinted genes in *B. napus* enriched the gene imprinting in dicotyledon plants, and provided a basis for further researches on how gene imprinting regulates seed development.

## Materials and methods

### Tissue collection and RNA preparation

Two *B. napus* L. cultivars (ZS11 and Y6) were provided by Jiangsu Institute of Agricultural Science in the Lixiahe District, and grown in the filed in Yangzhou, Jiangsu, China. Reciprocal crosses between two cultivars were carried out for F1 hybrids. The endosperm for RNA-seq was collected from immature seeds at 20 and 25 DAP, including the endosperm from reciprocal crosses and self-pollinated ZS11 and Y6. To avoid tissue contamination, the endosperm was sucked with an injector, which was inserted into the hole punched by a needle on the top of seeds (opposite to the embryo). Then, the endosperm was pooled and immediately stored in RNA extraction buffer [46]. Three biological replicates were included for each sample.

### RNA-seq analysis and data processing

Total RNA was extracted by RNAprep pure Plant Kit (TIANGEN, China). The purity, concentration, and integrity of endosperm RNA were detected using agarose gel electrophoresis, Nanodrop, Qubit, and Agilent 2100. The eligible RNA samples were used for mRNA library construction and high-throughput sequencing on the Illumina Novaseq 6000 platform [88]. In total, 269.84 Gb and 268.73 Gb of raw data were generated from parents and reciprocal hybrids endosperm, respectively. The sequencing base qualities and reads qualities were assessed by FastQC-0.11.8 software (http://www.bioinformatics.babraham.ac.uk/projects/fastqc/). Low-quality and adaptor sequences in the raw data were eliminated using Trimmomatic-0.36 software (http://www.usadellab.org/cms/uploads/supplementary/Trimmomatic/) [89]. Then, the clean data were retained for further analysis of SNP and gene expression. The correlation coefficients among the three biological replicates of each sample were assessed for data reliability (*R* > 0.95). The reads were mapped to the *B. napus* reference genome v4.1 (http://www.genoscope.cns.fr/brassicanapus/data/) using Hisat2–2.1.0 software (https://ccb.jhu.edu/software/hisat2/index.shtml) [90, 91], only two nucleotide mismatch was allowed in paired-end alignment. The mapping rate of all samples was > 90%.

### SNP calling and identification of imprinted genes

The RNA-seq data of ZS11 and Y6 were used for SNP screening. To increase the credibility, only the uniquely mapped reads were kept for analysis. SNP calling was performed using the Samtools-1.4 (http://samtools.sourceforge.net/) command Mpileup and the Bcftools program to identify the SNPs between ZS11 and Y6 [92, 93], and only the homozygous SNPs existed in at least two biological replicates and supported by ≥10 reads in each library were retained as parental specific SNPs.

To identify the imprinted genes, the uniquely mapped reads from reciprocal hybrid endosperm were retained for SNP and expression analysis. The sequencing reads of hybrid endosperm containing maternal-derived or paternal-derived SNPs were identified and counted by Perl scripts. Theoretically, the ratio of maternal-derived allele to paternal-derived allele in the hybrid endosperm should be 2: 1. We performed a two-tailed χ^2^ test on the ratio of maternal versus paternal allele counts for each gene in ZS11 (♀) × Y6 (♂) and Y6 (♀) × ZS11 (♂). Genes with parental allele bias deviated from 2: 1 in three biological replicates of both reciprocal hybrids were screened and defined as potential imprinted genes. We used stringent criteria to screen imprinted gene. Genes with a ratio of maternal-derived reads to paternal-derived reads ≥10: 1 (5 times of maternal: paternal = 2: 1, ≥ 90% maternally biased expression) in both reciprocal hybrids were taken as MEGs, while genes with a ratio of paternal-derived reads to maternal-derived reads ≥3: 2 (3 times of paternal: maternal = 1: 2, ≥ 60% paternally biased expression) were defined as PEGs. All the imprinted genes were screened with a threshold of *q* < 0.05.

### Reverse transcription and locus-specific sequencing

RNA from the endosperm of parents and reciprocal hybrids were used for confirmation of imprinted genes. The cDNA was synthesized using HiScript III RT SuperMix for qPCR (Vazyme, China). Primers used for sequence amplification were designed with Primer Premier 5.0 and listed in Additional file 12: Table S11. The amplified fragments from parents and reciprocal hybrids were sequenced by Sanger sequencing (TsingKe Biological Technology, China), to confirm the existence of maternal or paternal specific SNPs in MEGs or PEGs.

### Gene expression analysis

To investigate the expression pattern of imprinted genes in different tissues of *B. napus*, three biological replicates of root, stem, leaf, bud, and endosperm were collected for RNA-seq analysis. The sequencing data was mapped as mentioned above and the mapped reads were normalized using FPKM value. The heat map of imprinted genes in different tissues was generated based on the log_10_ transformed values of FPKM. The expression of imprinted and non-imprinted genes in parental endosperm were plotted with log_2_FPKM.

### Gene ontology analysis

GO enrichment was analyzed using OmicShare website (http://www.omicshare.com/tools/) and Blast2GO software (https://www.blast2go.com/) with a corrected *p* < 0.05. Samtools-1.4 was adopted for visualization of nucleic acid sequences based on the IDs of imprinted genes, and the output files were used for GO annotation with Blast2GO. Biomart tool (http://plants.ensembl.org/biomart/martview/) was used for preparation of input files for Omicshare website.

### Clustering analysis

For clustering analysis, we mapped 245 imprinted genes onto the 19 *B. napus* chromosomes (except for 52 candidate imprinted genes located in Ann_random and Cnn_random chromosomes) using MG2C (http://mg2c.iask.in/mg2c_v2.0/). To analyze the gene distribution in chromosomes, sliding windows of 1 Mb with step size 0.1 Mb were used to compare the number of mapped reference genes and these imprinted genes were classified into clusters (*p* < 0.05) for further analysis.

### TE enrichment analysis nearby the imprinted genes

To verify the relationship between the imprinted genes and TEs, we used TransposonPSI software (https://sourceforge.net/projects/transposonpsi/) and Bioperl::SearchIO module (http://www.bioperl.org/wiki/Installing_BioPerl) to analyze the type and number of TEs within 5 kb of the upstream and downstream of the imprinted genes and non-imprinted genes.

## Supplementary Information

**Additional file 1: Fig. S1**. Personal correlation coefficient analysis of three biological replicates of RNA-seq data.

**Additional file 2: Table S1**. SNPs between 20 DAP endosperm of Y6 and ZS11.

**Additional file 3: Table S2**. SNPs between 25 DAP endosperm of Y6 and ZS11.

**Additional file 4: Table S3**. The proportion of maternal and paternal reads in 20 DAP endosperm of reciprocal hybrids.

**Additional file 5: Table S4**. The proportion of maternal and paternal reads in 25 DAP endosperm of reciprocal hybrids.

**Additional file 6: Table S5**. Imprinted genes identified in 20 DAP endosperm of reciprocal hybrids.

**Additional file 7: Table S6**. Imprinted genes identified in 25 DAP endosperm of reciprocal hybrids.

**Additional file 8: Table S7**. Comparison of imprinted genes between 20 DAP and 25 DAP endosperm of reciprocal hybrids.

**Additional file 9: Table S8**. Tissue expressional pattern of imprinted genes (FPKM).

**Additional file 10: Table S9**. Cluster analysis of imprinted genes on *B. napus* chromosomes.

**Additional file 11: Table S10**. GO enrichment analysis of imprinted genes.

**Additional file 12: Table S11**. Primers used for validation of imprinted genes.

## Data Availability

All the data pertaining to the present study have been included in the tables and figures of the manuscript, and the authors are pleased to share all the data and plant materials upon reasonable request.

## Abbreviations

MEG: Maternal expressed gene
PEG: Paternal expressed gene
TE: Transposable element
*AGL*: *AGAMOUS-LIKE* MADS-box gene
ZS11: Zhongshuang 11
Y6: Yangyou 6
SNP: Single nucleotide polymorphism
DAP: Days after pollination
FPKM: Fragments per kilobase per million
KEGG: Kyoto encyclopedia of genes and genomes
GO: Gene ontology
FUT2: Xyloglucan fucosyltransferase 2
DMR: Differentially methylated region
