# Supplementary figures and images for: Genomic imprinted genes in reciprocal hybrid endosperm of Brassica napus
Source: BMC Plant Biol. 2021 Mar 16;21:140. doi: 10.1186/s12870-021-02908-8 (PMC7968328; doi:10.1186/s12870-021-02908-8)

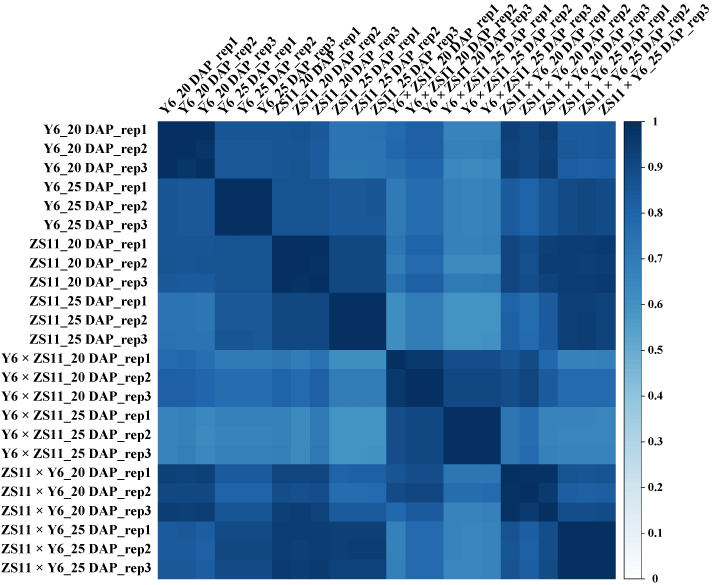

Supplement: Supplementary file 1 — Additional file 1: Fig. S1. Personal correlation coefficient analysis of three biological replicates of RNA-seq data. [file 12870_2021_2908_MOESM1_ESM.jpg]
